# Development of a triplex crystal digital RT-PCR for the detection of PHEV, PRV, and CSFV

**DOI:** 10.3389/fvets.2024.1462880

**Published:** 2024-12-12

**Authors:** Kaichuang Shi, Xin Hu, Yanwen Yin, Yuwen Shi, Yi Pan, Feng Long, Shuping Feng, Zongqiang Li

**Affiliations:** ^1^School of Basic Medical Sciences, Youjiang Medical University for Nationalities, Baise, China; ^2^College of Animal Science and Technology, Guangxi University, Nanning, China; ^3^Guangxi Center for Animal Disease Control and Prevention, Nanning, China

**Keywords:** porcine hemagglutinating encephalomyelitis virus (PHEV), porcine pseudorabies virus (PRV), classical swine fever virus (CSFV), multiplex crystal digital RT-PCR, detection method

## Abstract

Porcine hemagglutinating encephalomyelitis virus (PHEV), porcine pseudorabies virus (PRV), and classical swine fever virus (CSFV) are currently prevalent worldwide and cause similar neurological symptoms in infected pigs. It is very important to establish a detection method that can rapidly and accurately detect and differentiate these three viruses. Targeting the PHEV N gene, PRV gB gene, and CSFV 5′ untranslated region (5′UTR), three pairs of specific primers and probes were designed, and a triplex crystal digital reverse transcription-PCR (cdRT-PCR) was developed to detect PHEV, PRV, and CSFV. The results indicated that this assay had high sensitivity, and the limitation of detection (LODs) for PHEV, PRV, and CSFV were 4.812, 4.047, and 5.243 copies/reaction, respectively, which was about 50 times higher than that of multiplex real-time quantitative RT-PCR (RT-qPCR). This assay showed good specificity, without cross-reaction with other important swine pathogens, i.e., FMDV, PRRSV, PEDV, SIV, TGEV, PoRV, and PCV2. This assay had high repeatability, with intra-assay coefficients of variation (CVs) of 0.73–1.87%, and inter-assay CVs of 0.57–2.95%. The developed assay was used to test 1,367 clinical tissue samples from Guangxi province in China, and the positive rates of PHEV, PRV, and CSFV were 3.44% (47/1,367), 1.24% (17/1,367), and 1.90% (26/1,367), respectively, with a coincidence rate of 98.98% and a Kappa value of 0.94 to the reference multiplex RT-qPCR. The established triplex cdRT-PCR was a highly rapid, sensitive, and accurate assay to detect and differentiate PHEV, PRV, and CSFV.

## 1 Introduction

Porcine hemagglutinating encephalomyelitis virus (PHEV), pseudorabies virus (PRV), and classical swine fever virus (CSFV) can invade the brain of pigs and show similar neurological symptoms in infected pigs. They are difficult to distinguish depending only on the clinical symptoms in some cases, so accurate and reliable laboratory testing is necessary for differential diagnosis of these diseases.

PHEV was the earliest porcine coronavirus identified and the only known porcine neurotropic coronavirus (1). PHEV, a member of the β-coronavirus in the *Coronaviridae* family, is a positive-sense single-stranded RNA virus. Besides the special structure of the hemagglutinin-esterase protein (HE), PHEV contains four structural proteins, including surface spike glycoprotein (S), transmembrane glycoprotein (M), nucleocapsid protein (N), and membrane protein (E) (1). Porcine hemagglutinating encephalomyelitis (PHE) was first reported in Ontario, Canada in 1957, and those piglets infected with PHEV showed vomiting, anorexia, constipation, and severe progressive weight loss (2). Later, it was systematically reported that the infected newborn piglets developed anorexia, trembling, curling, and vomiting after 6–7 days old, followed by ataxia, hyperactivity, slapping, and other neurological symptoms, and died on 2–3 days post the onset of clinical symptoms (3). For grower and adult pigs, PHEV infection is subclinical because they can produce strong humoral immune response against PHEV, while for newborn piglets, PHEV infection is fatal (4, 5). Since PHE was first discovered in Canada in 1957, it has been reported in Europe, America, and Asia (1, 5–8). In China, PHE was first discovered in 1986, and the epidemic of PHEV has been reported since 2011 (8, 9). A large-scale epidemiological surveillance confirmed the prevalence of PHEV in at least eight provinces in southeastern China (10).

PRV, also called Suid herpesvirus I and Aujeszky virus, which belongs to the genus *varicella virus* of *herpesviridae* family, is an enveloped, double-stranded DNA virus (11). PRV consists of four protein structures, including linear DNA genome, capsid protein, tegument protein, and envelope protein. Ruminants, rodents, and predators can be infected by PRV, and pigs are the natural hosts and potential carriers (12, 13). Usually, the infected adult pigs show respiratory symptoms, while the infected piglets develop neurological symptoms (12). At present, PRV is still epidemic in many countries. Vaccination is the most effective measure to prevent and control the disease and minimize its economic loss (14, 15). However, due to the emergence of PRV variants in recent years, it is very hard to completely eradicate PR in many countries (16). In China, the first case of PRV infection was found in cats in 1947, and PRV was later found in pigs and other mammals (15). In recent years, PRV variants have been frequently reported in vaccinated pig farms in different provinces of China, which resulted in high mortality rate in newborn piglets (17, 18). The human case of endophthalmitis caused by PRV infection in 2017 indicated its zoonotic potential (19).

CSFV, which belongs to the *Pestivirus* genus in the *Flaviviridae* family (20), is a positive-sense single-stranded RNA virus, and cause a highly contagious venereal disease. A single open reading frame (ORF) is surrounded by 5′UTR and 3′UTR. CSFV contains four structural proteins, including core protein (C), envelope glycoprotein (E^rns^), and envelope glycoproteins (E1 and E2) (20, 21). Pigs are the only natural host of CSFV, and classical swine fever (CSF) has a serious impact on both domestic and wild pigs (21). CSF is characterized by high fever, loss of appetite, lethargy, vomiting, respiratory tract, digestive tract, and nervous system symptoms (21, 22). CSF was first discovered in the central and southern parts of the United States in the 1810s, and the earliest report on CSF in Europe was in England in 1862 (22). CSF has spread all over the world since the 1960s (21). At present, CSF is prevented mainly through biosecurity and vaccination (23, 24). Although many countries in North America, Oceania, and Europe have eradicated CSF, it is still prevalent in other regions (21, 25).

Digital PCR (dPCR) is a relatively new detection method that can quantify the target nucleic acid. The sample is first divided into many independent PCR sub-reactions, so each partition contains a small amount of target sequence or does not contain the target sequence, and each is amplified by PCR. After amplification, the presence or absence of fluorescence signal of each reaction unit is collected. Finally, the original absolute concentration is determined based on Poisson statistics (26). Compared with qPCR, dPCR has the highlight advantages as follows: it can quantify nucleic acids without reliance on external standards, standard curves, and Ct values; it can divide samples and amplify single molecule with higher accuracy and lower coefficient of variation (CV) value; it has higher tolerance to inhibitors than that of qPCR (26, 27); it is suitable for the detection of feces, sputum, and tissues known to contain a variety of inhibitors (food residues, bacteria, polysaccharides, etc.) (28, 29). Currently, two distinct approaches were available to perform dPCR, namely chamber digital PCR (cdPCR) and droplet digital PCR (ddPCR). The former relies on 2D arrays of microchambers to partition the sample (30), and the later partitions the sample in a bulk emulsion of microdroplets using platform-specific consumables (31). The Nacia system, which has three-color multiplexing capacity, was developed by Stilla Technologies (Villejuif, France) using a hybrid approach to perform dPCR. The approach combines the 2D array format of cdPCR and the use of droplet partitions as implemented in ddPCR (32). To date, a duplex ddPCR was established for the detection of PRV wild-type virus and gE-deleted vaccine strain (33), and a triplex cdPCR for the simultaneous detection of ASFV, CSFV, and PRRSV was established (34). However, there no report on using dPCR technology to simultaneously detect PHEV, PRV, and CSFV. In this study, a triplex cdRT-PCR targeting PHEV N gene, PRV gB gene, and CSFV 5′UTR was established using the Nacia system to distinguish these three pathogens. A total of 1,367 clinical tissue samples were used to validate the application of the established triplex cdRT-PCR.

## 2 Materials and methods

### 2.1 Reference viruses and clinical samples

The vaccine strains, including O/Mya98/XJ/2010 strain of foot-and-mouth disease virus (FMDV), C strain of CSFV, Bartha-K61 strain of PRV, CH-1R strain of porcine reproductive and respiratory syndrome virus (PRRSV), CV777 strain of porcine epidemic diarrhea virus (PEDV), TJ strain of swine influenza virus (SIV), H strain of porcine transmissible gastroenteritis virus (TGEV), NX strain of porcine rotavirus (PoRV), and WH strain of porcine circovirus type 2 (PCV2) were purchased from Huapai Biological Group (Chengdu, China). The vaccine strains of CSFV (WH-09, and CVCC AV1412 strains), and PRV (HB-2000, HN201, and EA strains) were purchased from Wuhan Keqian Biology Corporation Limited (Wuhan, China). The PHEV positive samples were provided by our laboratory. These vaccine viruses and positive samples were stored at −80°C until use.

From March 2023 to December 2023, 1,367 tissue samples were collected from slaughter pigs in slaughterhouses, and abnormal dead pigs in pig farms and harmless treatment plants in Guangxi province, southern China. The tissue samples from each pig included brain, lung, spleen, lung, and kidney, and the tissue homogenate of each pig was considered as one sample for testing viral nucleic acids. After collection, the samples were transported to the laboratory within 8 h under ≤ 4°C condition, and stored at −80°C until use.

### 2.2 Design of primers and probes

**Figure 1:**
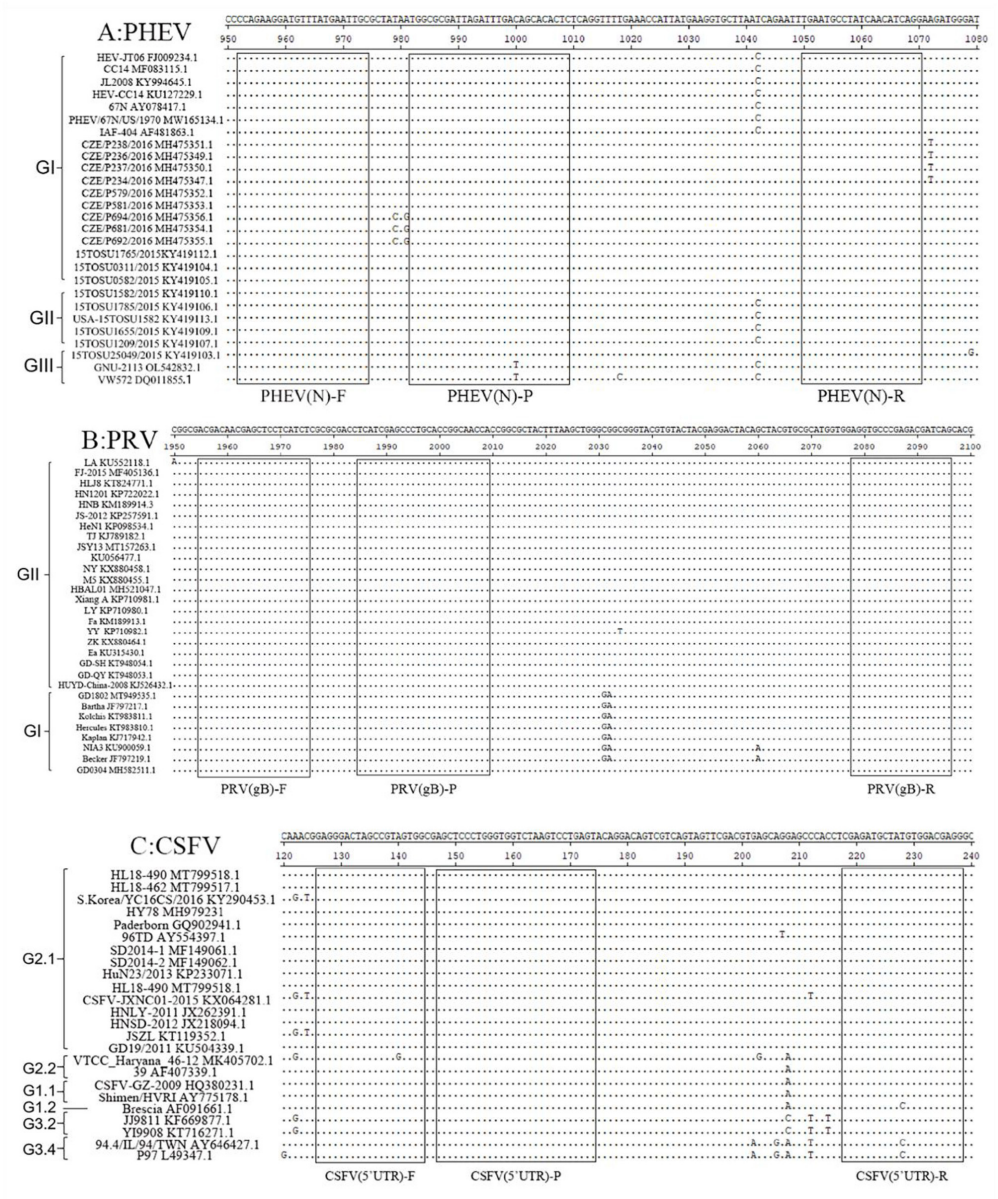
Primer and probe locations of the triplex cdRT-PCR. The locations of primers and probes are showed in the nucleotide sequence alignments of PHEV N gene **(A)**, PRV gB gene **(B)**, and CSFV 5′UTR **(C)**. F, P, and R indicate the forward primer, TaqMan probe, and reverse primer, respectively.

The genome sequences of PHEV, PRV, and CSFV were downloaded from National Center for Biotechnology Information (NCBI) (https://www.ncbi.nlm.nih.gov/nucleotide/, accession on 6 December 2021), and aligned. The conserved regions of PHEV N gene, PRV gB gene, and CSFV 5′UTR were selected as targeted regions (Figure 1), and three pairs of primers and probes were designed using Oligo 7.0 software (https://www.oligo.net/doenlods.html) (Table 1), as described by Hu et al. (35).

**Table 1:** The used primer and probe sequences for the triplex cdRT-PCR.

**Name**	**Sequence (5^′^ → 3^′^)**	**Tm/°C**	**Product/bp**
PHEV (N)-F	CCAGAAGGATGTTTATGAATTGC	54.1	119
PHEV (N)-R	CCTGATGTTGATAGGCATTCA	54.2	
PHEV (N)-P	FAM-TGGCGCGATTAGATTTGAYAGCACACTC-BHQ1	67.4	
PRV (gB)-F	ACGACAACGAGCTCCTCATCT	62.0	142
PRV (gB)-R	CTGATCGTCTCGGGCACCT	61.1	
PRV (gB)-P	VIC-TCATCGAGCCCTGCACCGGCAACCA-BHQ1	69.9	
CSFV (5′U)-F	GAGGGACTAGCCGTRGTGG	59.0	113
CSFV (5′U)-R	CCTCGTCCACRTAGCATCTCG	58.9	
CSFV (5′U)-P	CY5-AGCTCCCTGGGTGGTCTAAGTCCTGAGT-BHQ2	60.9	

### 2.3 Extraction of nucleic acids

The clinical tissue samples (0.5 g of brain, lung, spleen, and kidney each) were put into the 2.0 mL tube, and then 1.0 mL phosphate buffer solution (PBS, pH 7.2) and an aseptic steel ball were added to the tube. Then ground in an oscillating grinder for 5 min, frozen and thawed 3 times, and then centrifuged at 4°C (12,000 rpm for 5 min) to obtain the supernatants. The total viral RNA and DNA was extracted from 200 μL supernatants using MiniBEST Viral RNA/DNA Extraction Kit Ver.5.0 (TaKaRa, Dalian, China), and stored at −80°C until use.

### 2.4 Construction of standard plasmids

The PRV DNA (extracted from vaccine strain), and the PHEV, and CSFV cDNA (RNAs extracted from PHEV-positive sample and CSFV vaccine strain were reverse transcribed into cDNAs) were used to amplify the target fragments using PCR with the specific primers (Table 1). PCR products were added into 1% agarose gel, and put into an electrophoretic apparatus with 1× TAE buffer for 120 V gel electrophoresis for 30 min. At the end of electrophoresis, the target fragments were observed using the UVItec fluorescent analysis system (Cambridge, United Kingdom). The amplification products were purified using MiniBEST DNA Fragment Purification Kit Ver.4.0 (TaKaRa, Dalian, China), cloned into pMD18-T vectors (TaKaRa, Dalian, China), then transformed into *E. coli* DH5α cells (TaKaRa, Dalian, China). The DH5α cells and SOC medium were mixed together, and oscillating cultured at 37°C, 225 rpm for 1 h. One hundred microliters of cultured medium were inoculated in LB nutrient agar medium containing ampicillin and were cultured at 37°C for 12–14 h. The positive colony was selected and inoculated in an LB broth medium containing ampicillin and cultured at 37°C for 12 h. Then, 100 mL bacterial liquid was added to a 3,900 mL LB broth medium containing ampicillin and cultured at 37°C for 12–14 h in a constant temperature shaker. Then, the plasmid constructs were extracted from the cultured bacterial liquid using MiniBEST Plasmid Purification Kit Ver.4.0 (TaKaRa, Dalian, China). The plasmid constructs were sent to Aiji Biology Corporation Limited (Guangzhou, China) for sequencing, and confirmed by BLAST analysis at NCBI (https://www.ncbi.nlm.nih.gov/). The obtained recombinant plasmid constructs were named p-PHEV, p-PRV, and p-CSFV, respectively, and used as standard plasmids in this study. The ultraviolet absorbance and concentration X (ng/μL) at 260 and 280 nm wavelengths were measured using NanoDrop spectrophotometer (Thermo Fisher, Waltham, MA, USA). The plasmid constructs were calculated for their concentrations according to the formula below, diluted to 1.0 × 10^9^ copies/μL, and stored at −80°C until use.


$$\begin{array}{l} plasmid\;\left(copies/\mu L\right)=\frac{\left(6.02\times 10^{23}\right)\times \left(X\;ng/uL\times 10^{-9}\right)}{plasmid\;length\;\left(bp\right)\times 660} \end{array}$$

### 2.5 Determination of reaction conditions

The reaction system of triplex cdRT-PCR contained 12.5 μL 2× PerfeCTa Multiplex qPCR Tough Mix (Cycloud, Beijing, China), 2.5 μL Fluorescein Sodium Salt (Cycloud, Beijing, China), 2.5 μL primer and probe, 2.5 μL standard plasmid mixture, and nuclease-free distilled water to a total volume of 25 volume. The optimal reaction concentrations of primers and probes were determined through testing on different concentrations (600–1,000 nM for primer, and 200–400 nM for probe). The optimal annealing temperature were determined through optimizing in the range of 55–60°C. The reaction procedure of triplex cdRT-PCR was as follows: 95°C for 5 min; 45 cycles of 95°C for 5 s, 55–60°C for 30 s, 72°C for 30 s; and 72°C for 5 min.

The 25 μL reaction system was added to the sample hole of the Sapphire Chip (Cycloud, Beijing, China), and the chip was placed into the Naica automatic droplet chip digital PCR system (Stilla Technologies^TM^, Villejuif, France) to perform the cdRT-PCR reaction. Then, the chip was transferred to Prism3 droplet reading and analyzer (Stilla Technologies^TM^, Villejuif, France) for three-color fluorescence imaging, which was the images of FAM (blue), VIC (green), and Cy5 (red) detection channels. Finally, the results of image amplification were analyzed in Crystal Reader acquisition and data analysis software (Stilla Technologies^TM^, Villejuif, France).

### 2.6 Generation of standard curves

The mixture with three plasmid constructs p-PHEV, p-PRV, and p-CSFV was continuously diluted 10-fold from 1.0 × 10^5^ to 1.0 × 10^1^ copies/μL (the final reaction concentration in the reaction system was 1.0 × 10^4^ to 1.0 × 10^0^ copies/μL), and the standard curves of the established cdRT-PCR were generated.

### 2.7 Analysis of specificity

The specificity analysis of triplex cdRT-PCR was done using the mixture of p-PHEV, p-PRV, and p-CSFV, the vaccine strains of FMDV, CSFV, PRRSV, PRV, PEDV, TGEV, PoRV, PCV2, and SIV, and the positive samples of PHEV, PRV, and CSFV. The nucleic acid of negative tissue, and nuclease-free distilled water were used as negative controls.

### 2.8 Analysis of sensitivity

The mixture of p-PHEV, p-PRV, and p-CSFV was diluted 10-flod from 1.0 × 10^6^ to 1.0 × 10^0^ copies/μL (the final reaction concentration: 1.0 × 10^5^ to 1.0 × 10^−1^ copies/μL), and used as templates to perform the cdRT-PCR. The LOD of the assay was determined based on Poisson distribution analysis.

In addition, the Probit regression analysis (https://www.ibm.com/cn-zh/spss) was also used to analyze the LODs of the assay in order to further verify the results of the Poisson distribution analysis. It was used to analyze the relationship between positive hit probability and detection concentration, to evaluate the sensitivity of this method. The mixture of p-PHEV, p-PRV, and p-CSFV was diluted 2-flod, i.e., 125, 62.5, 31.25, 15.625, 7.813, 3.906, 1.953, 0.977, and 0.488 copies/reaction, and used as templates. Each concentration was set for 20 repeats, and the times of positive amplification curve were counted. The results were analyzed using IBM SPSS Statistics 27 software (https://www.ibm.com/cn-zh/spss) and StataMP 17 software (https://www.stata.com/products/windows/).

### 2.9 Analysis of repeatability

The coefficiency variation (CV) values were used to analyze the repeatability of the triplex cdRT-PCR. The mixture of p-PHEV, p-PRV, and p-CSFV was diluted 10-flod from 1.0 × 10^5^ to 1.0 × 10^3^ copies/μL (final reaction concentration: 1.0 × 10^4^ to 1.0 × 10^2^ copies/μL), and used as templates. The cdRT-PCR was performed in triplicate to determine intra-assay CV, and performed on three different days to determine inter-assay CV.

### 2.10 Testing of clinical samples

The 1,367 clinical samples collected from Guangxi province from March 2023 to December 2023 were tested using the established triplex cdRT-PCR to evaluate the applicability of the assay. Furthermore, the 1,367 samples were also tested using a quadruplex RT-qPCR established by Hu et al. (35). The results were analyzed using IBM SPSS Statistics 27 software (https://www.ibm.com/cn-zh/spss), and the coincidence rates and Kappa values were calculated.

## 3 Results

### 3.1 Construction of standard plasmids

The amplification products of PHEV N gene, PRV gB gene, and CSFV 5′UTR were purified, and cloned to obtain the recombinant plasmid constructs, then sequenced. The sequences were confirmed by BLAST analysis in NCBI. The obtained standard plasmid constructs were named p-PHEV, p-PRV, and p-CSFV, respectively. Then, the concentrations of three plasmid constructs were determined to be 5.80 × 10^10^, 5.02 × 10^10^, and 6.94 × 10^10^ copies/μL, respectively. All plasmid constructs were diluted to 1.0 × 10^9^ copies/μL, and stored at −80°C until use.

### 3.2 Determination of reaction conditions

**Figure 2:**
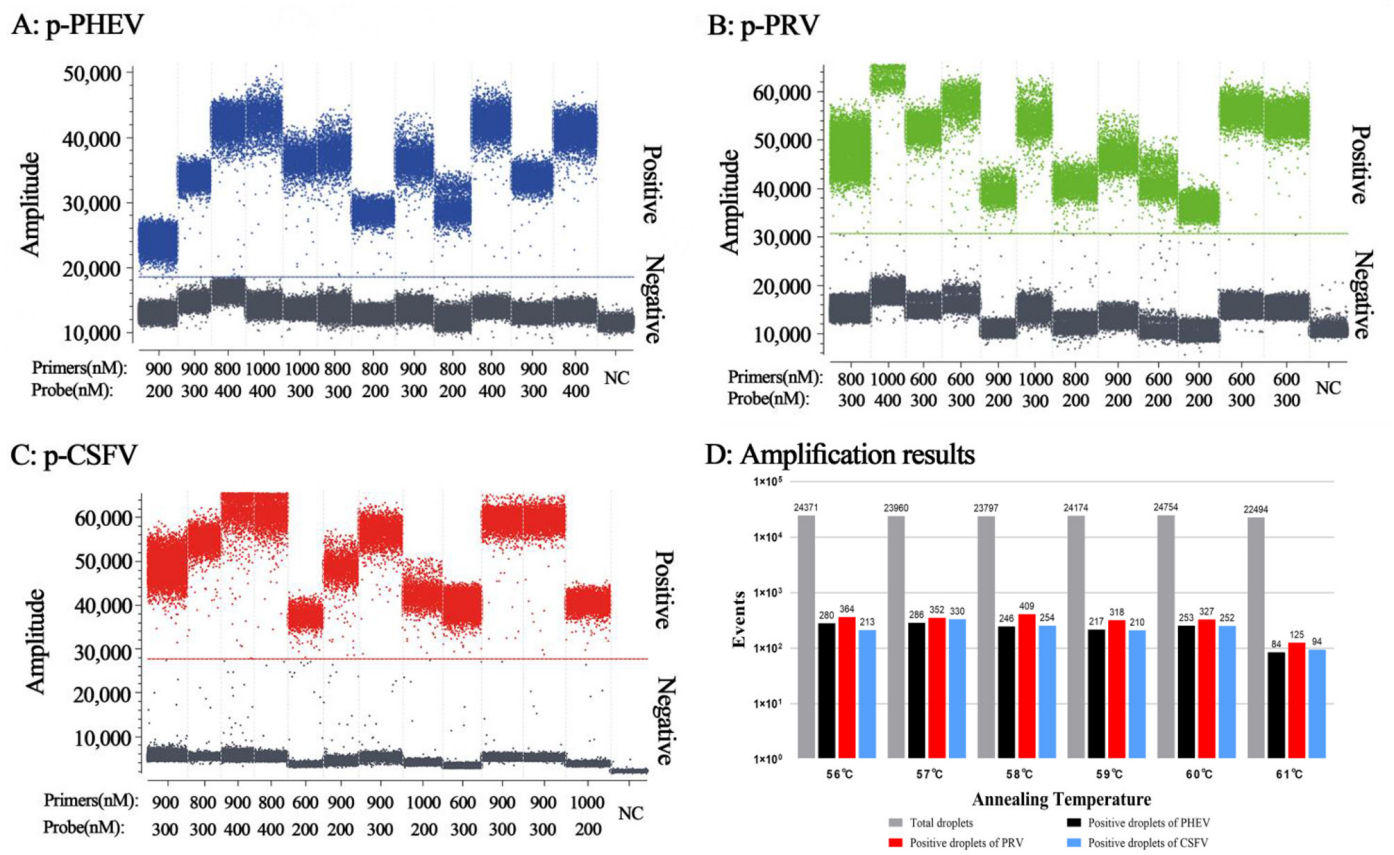
Determination of the primer and probe concentrations **(A–C)** and annealing temperatures **(D)** for the triplex cdRT-PCR. **(A–C)** The amplification results of p-PHEV, p-PRV, and p-CSFV plasmid constructs (final reaction concentrations: 1.0 × 10^4^ copies/μL) with different concentrations of probes and primers. D, the amplification results at different annealing temperatures; NC, negative control.

The optimal reaction conditions included the optimal concentration of primers and probes, and the optimal annealing temperature. Based on the high fluorescence signal values of positive droplets, relatively concentrated droplets, obvious division of negative droplets and positive droplets, the high absolute concentration of positive droplets, and a small number of droplets diffused in the middle of triplex cdRT-PCR (Figures 2A–C), the optimal annealing temperature was determined to be 57°C (Figure 2D), and the optimal concentrations of primers and probes were determined (Table 2).

**Table 2:** The reaction system for the triplex cdRT-PCR.

**Regent**	**Volume (μL)**	**Final concentration (nM)**
PerfeCta Multiplex qPCR ToughMix (2×)	12.5	1 ×
Fluorescein Sodium Salt (1 μM)	2.5	100
PHEV (N)-F (25 μM)	0.9	900
PHEV (N)-R (25 μM)	0.9	900
PHEV (N)-P (25 μM)	0.3	300
PRV (gB)-F (25 μM)	0.9	900
PRV (gB)-R (25 μM)	0.9	900
PRV (gB)-P (25 μM)	0.2	200
CSFV (5′U)-F (25 μM)	1.0	1,000
CSFV (5′U)-R (25 μM)	1.0	1,000
CSFV (5′U)-P (25 μM)	0.2	200
Total nucleic acids	2.5	/
Nuclease-free distilled H_2_O	Up to 25	/

### 3.3 Generation of standard curves

**Figure 3:**
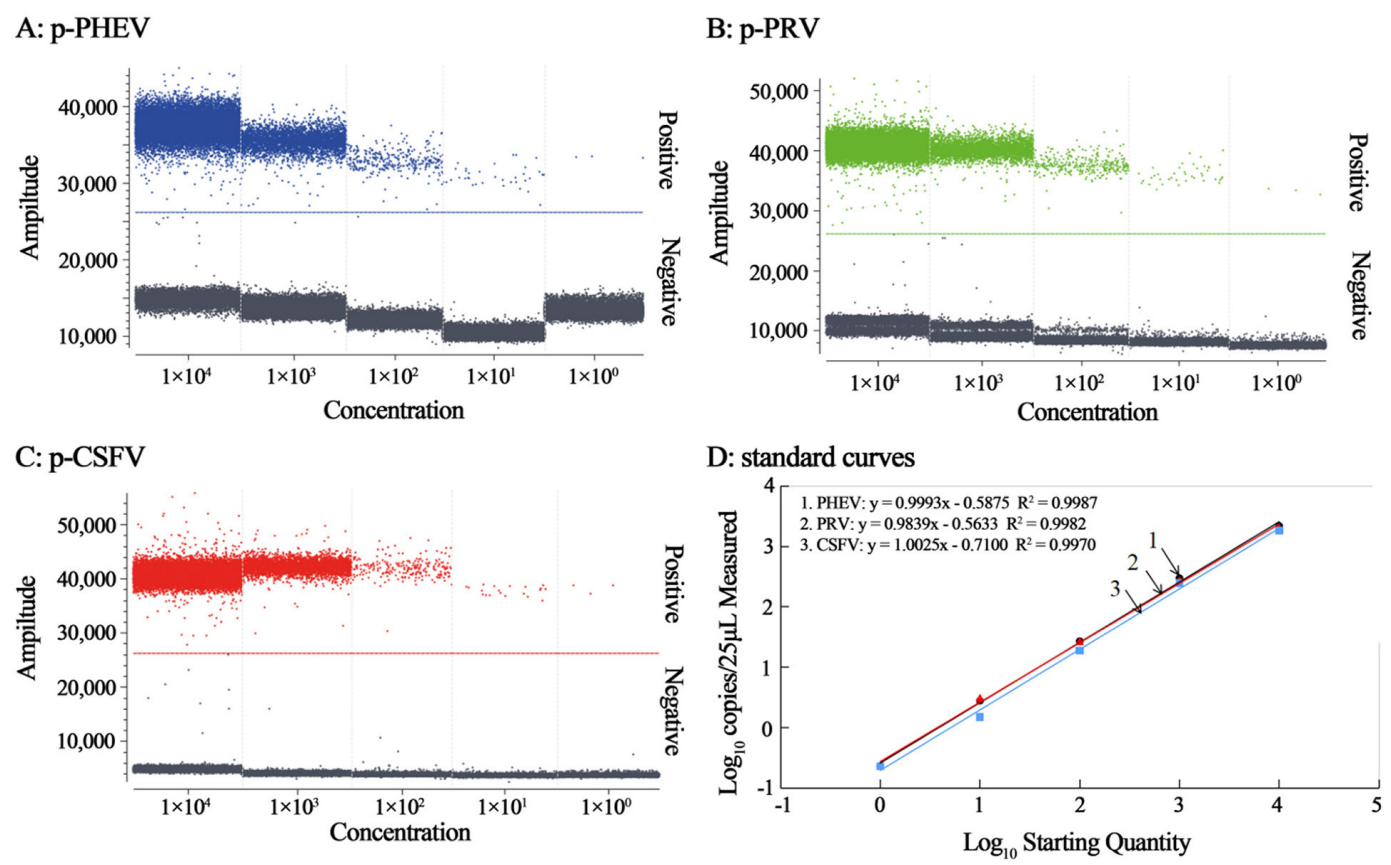
The amplification of p-PHEV, p-PRV, and p-CSFV (the final reaction concentration: 1.0 × 10^4^ to 1.0 × 10^0^ copies/μL) **(A–C)**, and the standard curves of the triplex cdRT-PCR **(D)**.

The mixtures of p-PHEV, p-PRV, and p-CSFV diluted from 1.0 × 10^5^ to 1.0 × 10^1^ copies/μL (the final reaction concentration: 1.0 × 10^4^ to 1.0 × 10^0^ copies/μL) were used as templates, and the cdRT-PCR amplification was carried out using the optimal reaction conditions to generate the standard curves (Figure 3). The results showed that the slope and correlation coefficient (*R*^2^) of the standard curves of PHEV, PRV, and CSFV were 0.9993 and 0.9987, 0.9839 and 0.9982, and 1.0025 and 0.9970, respectively (Figure 3D).

### 3.4 Analysis of specificity

**Figure 4:**
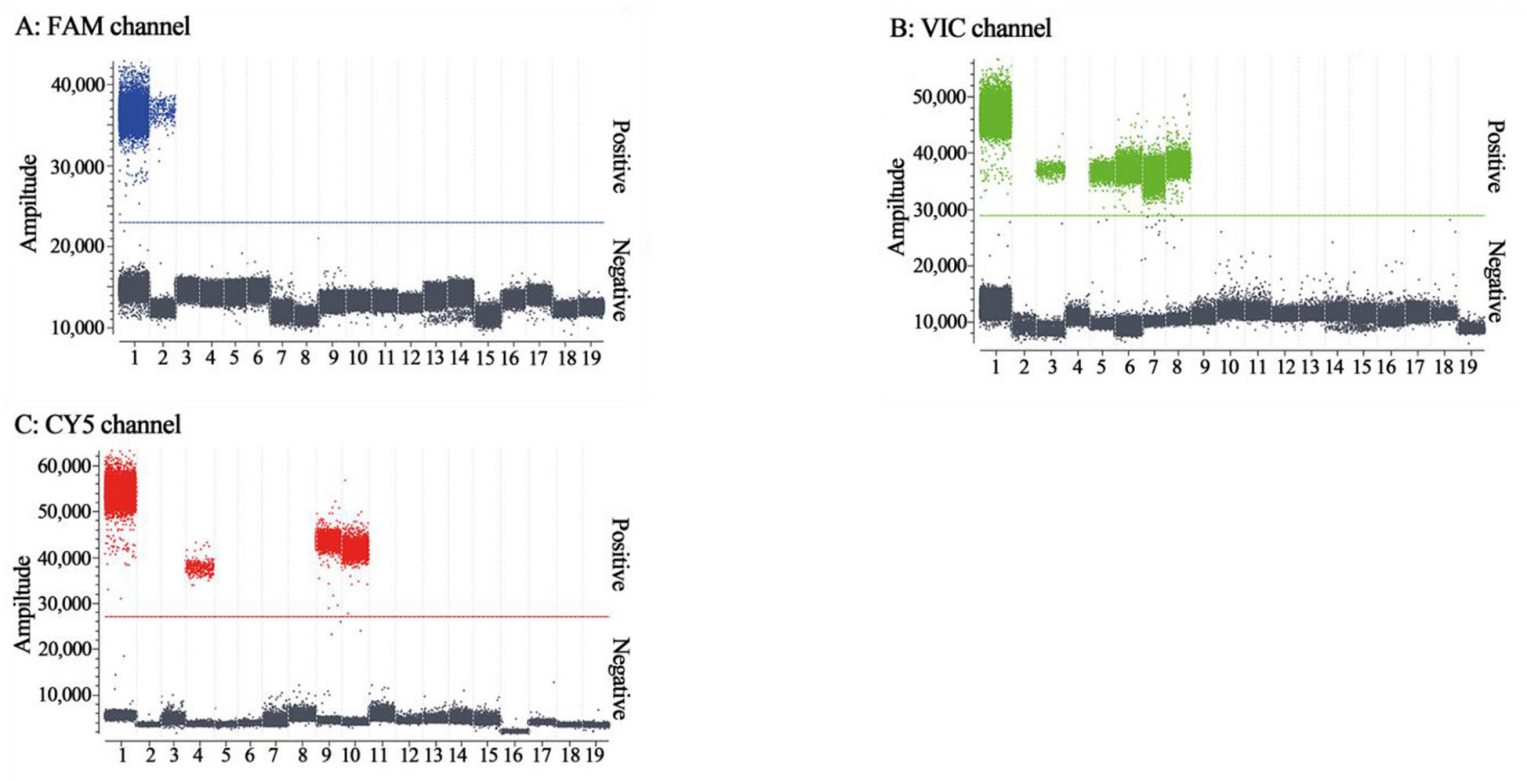
The specificity analysis of the triplex cdRT-PCR. **(A–C)** The amplification results of FAM channel for PHEV, VIC channel for PRV, and CY5 channel for CSFV. 1: the mixture of p-PHEV, p-PRV, and p-CSFV; 2-4: clinical positive sample of PHEV, PRV, and CSFV; 5: PRV Bartha-K61 strain; 6: PRV HB2000 strain; 7: PRV HN1201 strain; 8: PRV EA strain; 9: CSFV CVCC AV1412 strain; 10: CSFV WH-09 strain; 11–17: PEDV CV777 strain, TGEV H strain, PoRV NX strain, PRRSV CH-1R strain, PCV2 WH strain, FMDV O/Mya98/XJ/2010 strain, and SIV TJ strain; 18: Clinical negative tissue sample; 19: Nuclease-free distilled water as negative control.

The specificity of triplex cdRT-PCR was analyzed using the mixture of p-PHEV, p-PRV, and p-CSFV, and FMDV, PRRSV, PRV, PEDV, PHEV, TGEV, PoRV, PCV2, and SIV. The results showed that the triplex cdRT-PCR could detect only PHEV, PRV, and CSFV, and other viruses could not generate any fluorescence signal, indicating that this method has good specificity (Figure 4).

### 3.5 Analysis of sensitivity

**Figure 5:**
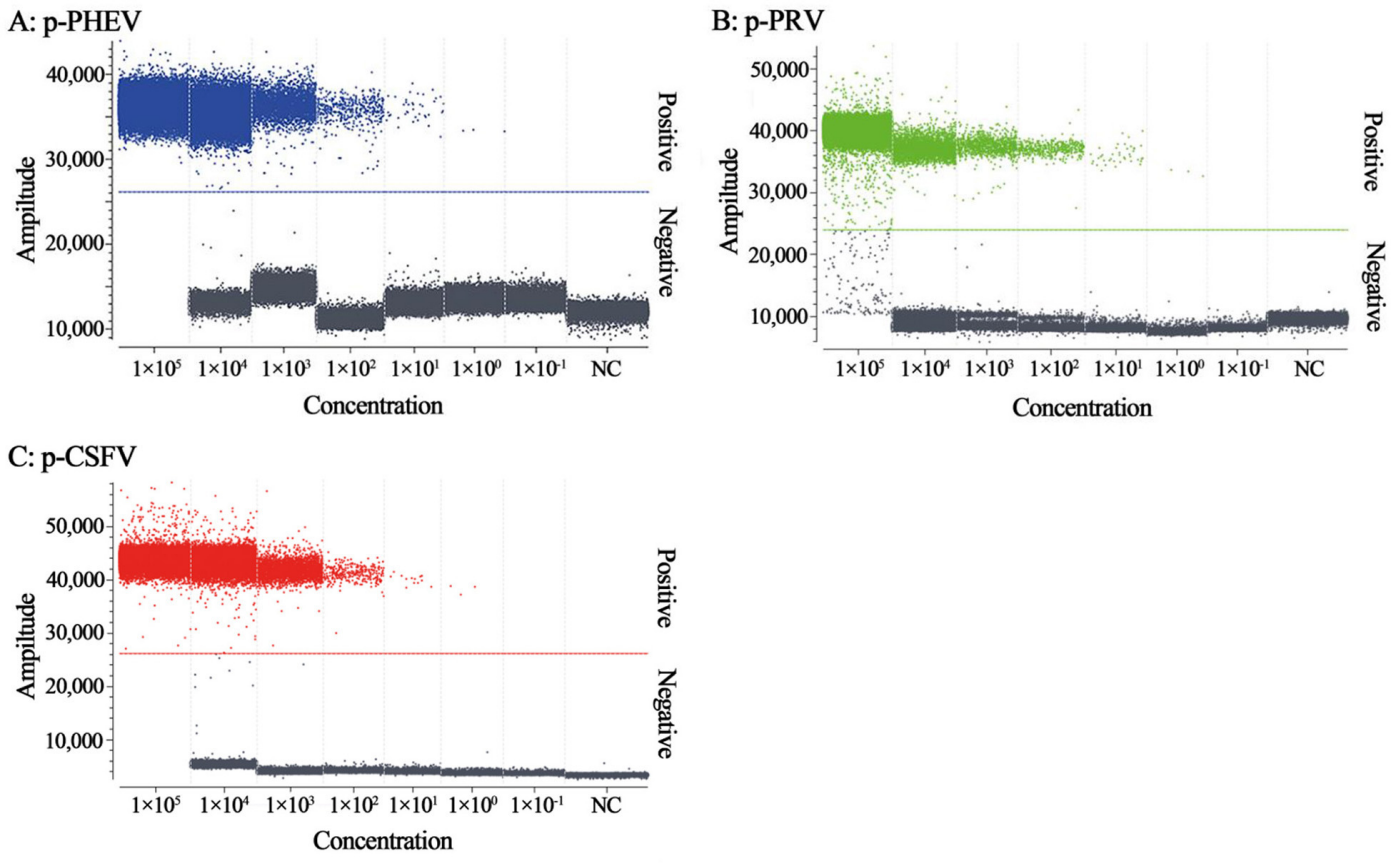
The sensitivity analysis of the triplex cdRT-PCR. The p-PHEV **(A)**, p-PRV **(B)**, and p-CSFV **(C)** mixtures range from 1.0 × 10^5^ to 1.0 × 10^−1^ copies/μL. NC, nuclease-free distilled water as negative control.

**Figure 6:**
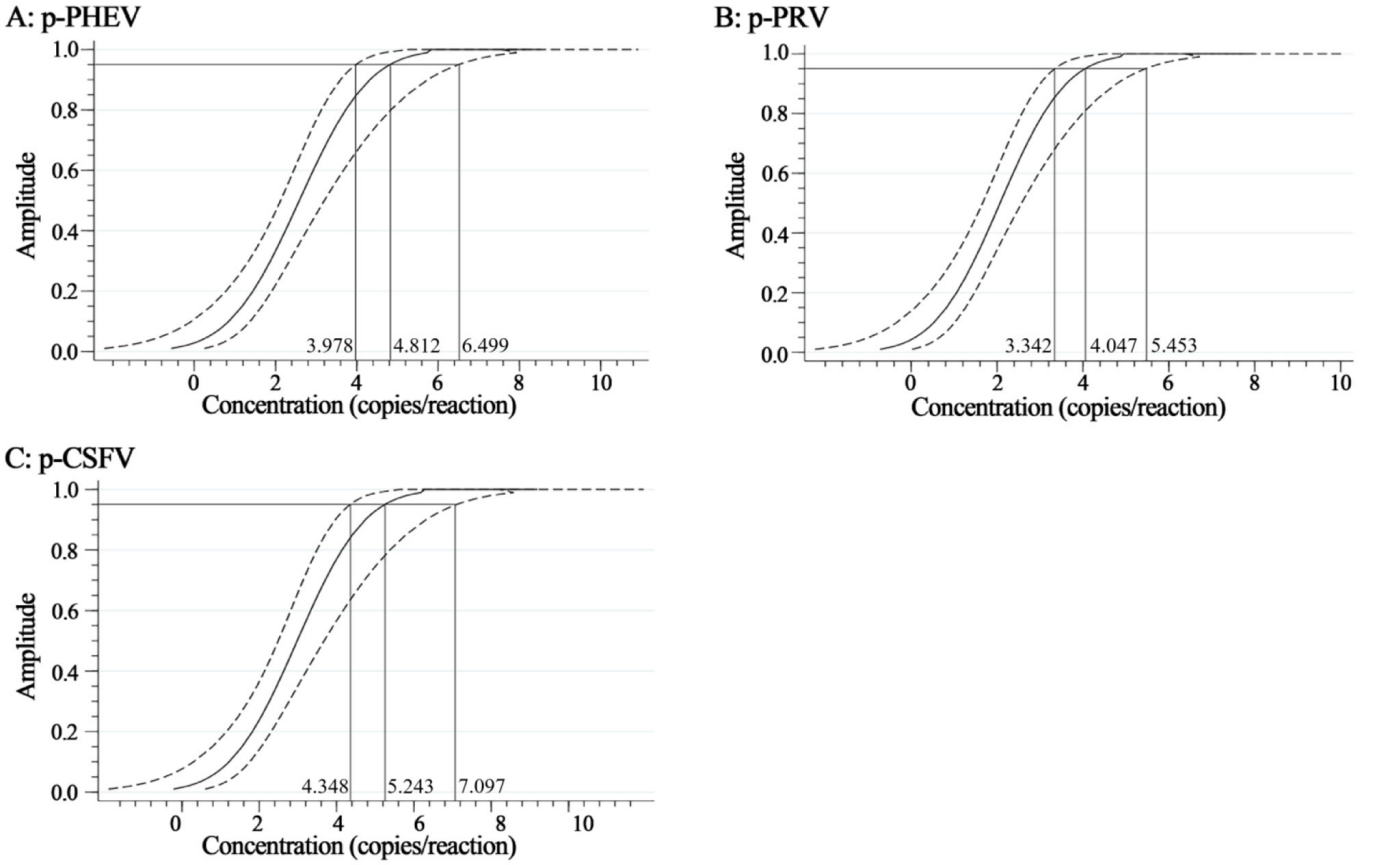
The sensitivity based on Probit regression analysis. At 95% confidence interval, the LODs of p-PHEV **(A)**, p-PRV **(B)**, and p-CSFV **(C)** were determined to be 4.812 (3.978–6.499), 4.047 (3.342–5.453), and 5.243 (4.348–7.097) copies/reaction, respectively.

The mixtures of p-PHEV, p-PRV, and p-CSFV diluted from 1.0 × 10^5^ to 1.0 × 10^−1^ copies/μL (final reaction concentration in the reaction system) were used as templates to evaluate the LOD of the triplex cdRT-PCR. Based on Poisson distribution analysis, the LODs of p-PHEV, p-PRV, and p-CSFV were determined to be 3.650, 3.025, and 4.000 copies/reaction, respectively (Figure 5). In addition, the sensitivity of this method was also evaluated using Probit probability regression analysis, and the positive hit rates were obtained (Table 3). The LODs of p-PHEV, p-PRV, and p-CSFV were determined to be 4.812 [3.978–6.499 at 95% confidence interval (CI)], 4.047 (3.342–5.453 at 95% CI), 5.243 (4.348–7.097 at 95% CI) copies/reaction, respectively (Figure 6).

**Table 3:** The hit rates for serial dilution of plasmid constructs.

**Name**	**Concentration (copies/reaction)**	**Number of samples**	**Positive samples**	**Hit rate (%)**
p-PHEV	15.63	20	20	100
	7.81	20	20	100
	3.91	20	16	80
	1.96	20	8	40
	0.98	20	3	15
	0.46	20	0	0
p-PRV	15.63	20	20	100
	7.81	20	20	100
	3.91	20	18	90
	1.96	20	11	55
	0.98	20	5	25
	0.46	20	0	0
p-CSFV	15.63	20	20	100
	7.81	20	20	100
	3.91	20	14	70
	1.96	20	7	35
	0.98	20	1	5
	0.46	20	0	0

### 3.6 Analysis of repeatability

The repeatability of the triplex cdRT-PCR was evaluated using three concentrations of p-PHEV, p-PRV, and p-CSFV (the final reaction concentration: 1.0 × 10^4^, 1.0 × 10^3^, and 1.0 × 10^2^ copies/μL). The results showed that the intra-assay CV and inter-assay CV were 0.73–1.87% and 0.57–2.95%, respectively, indicating that the method has excellent repeatability (Table 4).

**Table 4:** Repeatability analysis of the triplex cdRT-PCR.

**Plasmid**	**Concentration (copies/μL)**	**Intra-assay for repeatability (copies/reaction)**			**Inter-assay for repeatability (copies/reaction)**		
		$\bar{X}$	**SD**	**CV (%)**	$\bar{X}$	**SD**	**CV (%)**
p-PHEV	1.0 × 10^4^	59,033.33	946.81	1.60%	58,025.00	1,442.22	2.49%
	1.0 × 10^3^	5,945.00	96.44	1.62%	5,885.00	148.39	2.52%
	1.0 × 10^2^	505.83	6.29	1.24%	541.67	12.33	2.28%
p-PRV	1.0 × 10^4^	61,383.33	977.99	1.59%	59,966.67	1,038.73	1.73%
	1.0 × 10^3^	5,925.83	111.03	1.87%	5,866.67	82.70	1.41%
	1.0 × 10^2^	538.33	8.78	1.63%	560.83	10.10	1.80%
p-CSFV	1.0 × 10^4^	50,850.00	587.90	1.16%	49,808.33	1,176.95	2.36%
	1.0 × 10^3^	4,970.00	36.31	0.73%	5,030.00	28.83	0.57%
	1.0 × 10^2^	469.17	7.64	1.63%	472.50	13.92	2.95%

### 3.7 Detection results of clinical samples

**Figure 7:**
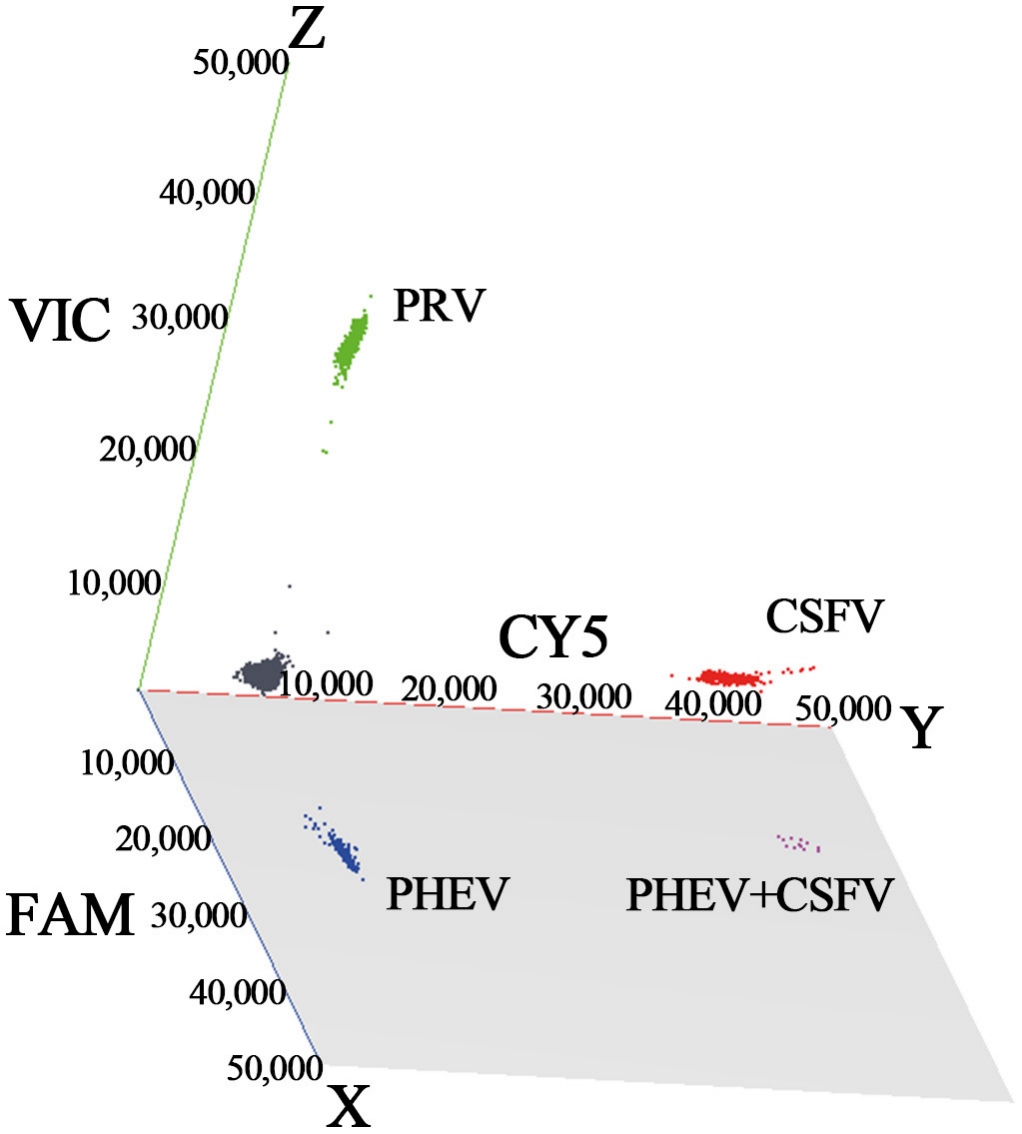
The detection results of the triplex cdRT-PCR. The 3D scatterplots of fluorescence intensities were acquired in the FAM for PHEV (*x*-axis), CY5 for CSFV (*y*-axis), and VIC for PRV (*z*-axis) acquisition channels.

The established triplex cdRT-PCR was used to detect 1,367 clinical tissue samples collected from different regions in Guangxi province during March 2023 and December 2023. The positive rates of PHEV, PRV, and CSFV were 3.44, 1.24, and 1.90%, respectively (Figure 7; Table 5). In addition, a quadruplex RT-qPCR established by Hu et al. (35) was also used to test these samples, and the results were analyzed by IBM SPSS Statistics. Their coincidence rates and Kappa values were showed in Table 6. Comparing the results of cdRT-PCR and RT-qPCR, it was found that at 95% confidence interval, the clinical sensitivities of PHEV, PRV, and CSFV were all 100%, and the clinical specificities of PHEV, PRV, and CSFV were 99.47% (98.92–99.74%), 99.78% (99.35–99.92%), and 99.70% (99.24–99.88%), respectively (Table 7). The coincidence rate of two methods was 98.98% (Table 8).

**Table 5:** Detection results of the clinical samples.

**Date**	**Number**	**Positive samples**		
		**PHEV (%)**	**PRV (%)**	**CSFV (%)**
Mar, 2023	24	4 (16.67%)	1 (4.17%)	3 (12.50%)
Apr, 2023	184	2 (1.09%)	0	11 (5.98%)
May, 2023	274	4 (1.46%)	0	8 (2.92%)
Jul, 2023	475	17 (3.58%)	13 (2.74%)	0
Aug, 2023	263	12 (4.56%)	0	2 (0.76%)
Sep, 2023	30	1 (3.33%)	0	0
Oct, 2023	60	4 (6.67%)	1 (1.67%)	2 (3.33%)
Dec, 2023	57	3 (5.26%)	2 (3.51%)	0
Total	1,367	47 (3.44%)	17 (1.24%)	26 (1.90%)

**Table 6:** Detection results of the clinical samples using the triplex cdRT-PCR and the reference RT-qPCR.

**Pathogen**	**Number**	**RT-qPCR**		**cdRT-PCR**		**Coincidence rate (%)**	**Kappa value**
		**Positive**	**Positive rate (%)**	**Positive**	**Positive rate (%)**		
PHEV	1,367	40	2.93	47	3.44	99.49	0.92
PRV	1,367	14	1.02	17	1.24	99.78	0.90
CSFV	1,367	22	1.61	26	1.90	99.71	0.92

**Table 7:** Clinical sensitivity and specificity of the triplex cdRT-PCR.

**cdRT-PCR**		**RT-qPCR**		**Total**	**Clinical sensitivity**	**Clinical specificity**
		**Positive**	**Negative**			
PHEV	Positive	40	7	47	100.00%	99.47%
	Negative	0	1,320	1,320		
	Total	40	1,327	1,367		
PRV	Positive	14	3	17	100.00%	99.78%
	Negative	0	1,350	1,350		
	Total	14	1,353	1,367		
CSFV	Positive	22	4	26	100.00%	99.70%
	Negative	0	1,341	1,341		
	Total	22	1,345	1,367		

**Table 8:** Comparison of the results using the triplex cdRT-PCR and the reference RT-qPCR.

**RT- qPCR**	**cdRT-PCR**			**Coincidence rate (%)**	**Kappa value**
	**Positive**	**Negative**	**Total**		
Positive	76	0	76	98.98	0.91
Negative	14	1,277	1,291		
Total	90	1,277	1,367		

## 4 Discussion

In a study on early polymerase chain reaction published in 1988 by Saiki et al. (36), digital polymerase chain reaction (dPCR) was proposed for the first time, while the concept of dPCR was first put forward by Kinzler and Vogelstein (37). Like qPCR, dPCR can qualitatively and quantitatively analyze the target molecule, but the difference between dPCR and qPCR is that in the process of dPCR reaction, the samples are cut into individual molecules, and then amplified by PCR to obtain all or none signal, which is the digital signal. The digital signal is used to analyze the properties of the target molecules and the number of target molecules can be calculated based on Poisson distribution analysis (38). Compared with qPCR, the most prominent advantage of dPCR is that dPCR is absolutely quantitative. The quantification in the qPCR reaction is based on the analysis of the fluorescence signal in the exponential stage, and the quantity of the target sequence is measured relative to the standard curve produced by the known number of standard samples, which means this method for quantifying the target sequence is based on the premise that the amplification efficiency of the sample and the standard sample are equal, so the difference in qPCR reaction efficiency will affect the accuracy of sample quantification (39, 40). In contrast, dPCR can truly quantify the target sequence in the sample. dPCR collects the fluorescence signal at the end of the reaction and uses the number of total positive regions to analyze and calculate the concentration of the target sample, which is an effective method for sample separation and single molecule target amplification (26). In other words, dPCR does not depend on the calibration standard curve to quantify the sample, it is an absolute nucleic acid quantitative method depending on fluorescence signal detection and binomial events (with or without fluorescence) (41). Compared with qPCR, dPCR shows higher sensitivity and more stable repeatability (42). dPCR calculates the absolute content of the target sample by counting positive holes directly, which provides better comparable results in different tests. Even if qPCR has been widely used in laboratories for the detection of different kinds of samples (serum, cerebrospinal fluid, and tissue, etc.), there are still some problems with its sensitivity, accuracy, and repeatability, especially in low-concentration template amplification, qPCR shows great instability. Due to the difference of template quality, PCR reaction efficiency, and experimental conditions, the qPCR data from different laboratories or clinical trials are not comparable. Absolute quantification of pathogens can provide more powerful information for understanding the disease. Therefore, the application of dPCR in clinical detection may be of great benefit to the correct diagnosis and effective treatment of the disease (43, 44). At the same time, the high sensitivity and precision of dPCR make it helpful to more accurately detect low pathogen load and detect rare point mutations in the context of wild-type sequences (45).

dPCR has been used to evaluate the copy numbers of viruses, bacteria, and parasites in various clinical specimens (42, 43, 46). PHEV, PRV, and CSFV have been epidemic in pig herds in most countries around the world, and cause neurological symptoms and encephalitis in pigs, which is not easy to identify. Various laboratory techniques have been developed for detection of these pathogens, of which dPCR is a very good detection method due to the advantages of high sensitivity and accuracy, tolerance to inhibitors, and needless of dependance on standard curves to quantify samples (32, 47, 48). As a newly laboratory detection technology, dPCR has been developed for detection of swine viral pathogens, such as ASFV (49–52), CSFV (34), PRRSV (53), PRV (33), atypical porcine pestivirus (APPV) (54), and Japanese encephalitis virus (55). However, no multiplex dPCR method that can simultaneously detect PHEV, PRV, and CSFV has ever been reported until now.

Therefore, a triplex cdRT-PCR for the detection of PHEV, PRV, and CSFV was established in this study. Firstly, the concentrations of primers and probes, and the annealing temperatures were optimized. Then, the specificity, sensitivity, and repeatability were evaluated. Finally, the established assay was used to test 1,367 clinical samples to evaluate its applicability. The results showed that this assay can specifically detect only PHEV, PRV, and CSFV, and had high sensitivity with the LODs of 3.650, 3.025, and 4.000 copies/reaction using Poisson distribution analysis for p-PHEV, p-PRV, and p-CSFV, respectively. In addition, the Probit probability regression analysis indicated the LODs of p-PHEV, p-PRV, and p-CSFV were 4.812, 4.047, and 5.243 copies/reaction, respectively, which further confirmed the high sensitivity of the established triplex cdRT-PCR. The sensitivity of a quadruplex RT-qPCR using the same primers and probes were 176.25, 155.41, 175.83 copies/reaction, respectively (35), indicating that the triplex cdRT-PCR was about 50 times higher sensitive than the multiplex RT-qPCR. The 0.73–1.87% intra-assay CV, and 0.57–2.95% inter-assay CV indicated high repeatability of this assay. The developed assay was used to test 1,367 clinical tissue samples, and the positive rates of PHEV, PRV, and CSFV were 3.44, 1.24, and 1.90%, respectively, with a total coincidence rate of 98.98%, and a Kappa value of 0.91 with the reference assay (35), indicating highly consistent between two methods. These results indicated that a specific, sensitive, repeatable, and accurate triplex cdRT-PCR for the detection of PHEV, PRV, and CSFV has been successfully developed in this study. Compared to the former reported cdPCR assays for the detection of wild-type and vaccine-type of PRV (duplex cdPCR) (33), or ASFV, CSFV, and PRRSV (triplex cdRT-PCR) (34), this is the first report on triplex cdRT-PCR for the sensitive, and accurate detection of PHEV, PRV, and CSFV in one reaction at the same time. Of course, the cdRT-PCR has the disadvantage of relatively high cost while compared to the RT-qPCR, but the development of multiplex cdRT-PCR decreases dramatically the average cost of each sample, therefore it has been applied in more and more laboratories now.

The 1,367 clinical tissue samples collected from different regions of Guangxi province were tested using this method. The positive rates of PHEV, PRV, and CSFV were 3.44, 1.24, and 1.90%, respectively, indicating that PHEV, PRV, and CSFV are still epidemic in Guangxi province. Since the adult pigs infected with PHEV usually show subclinical infections, its harm to the pig industry is usually ignored. However, this pathogen may cause death to the infected piglets under 4 weeks. Recently, a metavirome analysis revealed a high prevalence of PHEV in clinically healthy pigs in 13 provinces in China (56). In addition, even if PRV has effective gE-deleted vaccine and CSFV has effective C-strain vaccine, PR and CSF are still prevalent in many pig farms in different provinces in China (15, 57–59). The economic losses of these diseases cannot be ignored, and the rapid, accurate diagnosis of these diseases is vital for prevention and control them. Therefore, the triplex cdRT-PCR can be used as sensitive, specific, and accurate method for the detection of PHEV, PRV, and CSFV. Since the still prevalence of PHEV, PRV, and CSFV in some pig farms, the developed triplex cdRT-PCR provides a useful method for investigation and epidemiology of these viruses, especially for the samples with very low viral loads.

Unfortunately, at present, the equipment and reagents used for dPCR are still relatively expensive, which limits the widespread application of this detection method in clinical practice. The triplex cdRT-PCR established in this study utilized three pairs of specific primers and probes to simultaneously detect three pathogens within a single detection well in one reaction, which greatly reducing detection cost for each sample and promoting its application in veterinary laboratories. Moreover, with the widespread application of this technology, the cost of equipment and reagents will gradually decrease, which will help dPCR to be accepted and applied by more and more veterinary laboratories in the future.

## 5 Conclusion

A triplex cdRT-PCR with high sensitivity, specificity, and repeatability was successfully established, which can simultaneously detect PHEV, PRV, and CSFV in one reaction within 2 h. The detection results of clinical tissue samples in Guangxi province from March 2023 to December 2023 indicated that PHEV, PRV, and CSFV were still prevalent in pig herds in Guangxi province.

## Data Availability

The raw data supporting the conclusions of this article will be made available by the authors, without undue reservation.
